# Editorial: Health Financing and Spending in Low- and Middle-Income Countries

**DOI:** 10.3389/fpubh.2021.800333

**Published:** 2021-12-21

**Authors:** Mihajlo Jakovljevic, Zafer Çalışkan, Paula Odete Fernandes, Sulaiman Mouselli, Michael Ekubu Otim

**Affiliations:** ^1^Institute of Comparative Economic Studies, Hosei University, Tokyo, Japan; ^2^Department of Global Health Economics and Policy, University of Kragujevac, Kragujevac, Serbia; ^3^Department of Economics, Hacettepe University, Ankara, Turkey; ^4^Escola Superior de Tecnologia e Gestão, Instituto Politécnico de Bragança, Bragança, Portugal; ^5^Faculty of Business Administration, Arab International University, Daraa, Syria; ^6^Department of Health Services Administration, University of Sharjah, Sharjah, United Arab Emirates

**Keywords:** health financing, health spending, health insurance, LMICs, health care, health policy

The World Bank has introduced the Atlas method to determine in an econometrically valid way borderline thresholds for classifications of all national economies into low, middle, and high-income categories in an objective and predictable manner. Although this approach may be challenged to some extent, it has long served well in observation and predictions of long-term trends in GDP growth and national health expenditures (1–3).

The contribution of low- and middle-income countries (LMICs) share in total health expenditures as observed through the (4) Global Health Expenditure criteria has almost doubled in terms of purchase power parity (PPP) basis from only 20% of the global share in 1995 up to almost 40% in 2013 (5, 6). These fiscal flows have been well-documented *via* WHO's National Health Accounts database. This lengthy and rocky road forward for the LMICs contains many difficulties. A few core challenges include socioeconomic inequalities in medical care access and affordability, large out-of-pocket expenses, and vulnerabilities against catastrophic household expenditures. These challenges remain matters of grave concern in many LMICs (7).

Broad trends give far greater grounds for optimism, however, since networks of rural and suburban health care facilities are growing and strengthening. Preventive lifestyle interventions, provision of essential medicines, and spreading of cost-effective basic medical technologies, designated in WHO policy as “best buys” interventions, all contributed to exceptionally improved early childhood survival and extended life expectancy. Current circumstances in most LMICs are characterized by aging populations, rapid urbanization, and increased citizen expectations in terms of health insurance coverage. Prescription drugs consumption is still dominated by generic medicines, with brand name originals gradually taking root. Hospital sectors are state or publicly owned in most former and modern day centrally-planned socialist economies. By contrast, in some regions like the Middle East and North African (MENA) Arabic nations, Latin America, and free-market Far East Asian economies, hospital property structure is predominantly privately owned (1).

Rapidly developing world regions exhibit substantial heterogeneity in terms of historical legacy in health care establishments, provision, and financing, however, they all face a few core common challenges. Among them, epidemiological transition in morbidity and mortality structure is probably the most notable. The burden of infectious diseases, nutritional disorders, and traumatism are gradually being replaced by chronic non-communicable diseases (NCDs). Infections tend to be of acute clinical course, affect juvenile and elderly populations outside the labor market, and are mostly curable with contemporary medicine. NCDs are chronic, costly, lifetime illnesses demanding complex and expensive medical care. Another core issue is related to decreasing working ability, absenteeism, and premature mortality imposed by NCDs on the working population of society. This means that the national health systems face a double burden in terms of workload and fiscal flows face from these dual long-term epidemiology trends. LMICs national health systems have historically evolved to combat primarily communicable disorders and now they are facing much harsher challenges (8, 9).

The article entitled “*Associations Between Schemes of Social Insurance and Self-Rated Health Comparison: Evidence From the Employed Migrants in Urban China*” explored the relationship between social insurance without health insurance and self-rated health comparison (SRHC) among employed migrants in urban China. Its results pointed out that only two of the three social insurance schemes could improve SRHC among the employed migrants (Guan).

Onwujekwe et al. contributed an intriguing piece entitled “*Characteristics and Effects of Multiple and Mixed Funding Flows to Public Healthcare Facilities on Financing Outcomes: A Case Study From Nigeria*” providing proof of concept that multiple funding flows to public hospitals are beneficial as well as constraining to health providers.

Nguyen-Thi et al. conducted the exceptional original research study entitled “*Cost-Effectiveness of Gliclazide-Based Intensive Glucose Control vs. Standard Glucose Control in Type 2 Diabetes Mellitus. An Economic Analysis of the ADVANCE Trial in Vietnam*.” They have highlighted that in Vietnam, gliclazide-based IGC was cost-effective compared with SGC from a healthcare payer perspective, as defined in the ADVANCE study.

The following article entitled “*Achieving Sustainable Development Goals (SDGs) in Sub-Saharan Africa (SSA): A Conceptual Review of Normative Economics Frameworks*” has provided sound evidence that the Non-Welfarist framework ought to be adopted in order to improve priority setting in the SSA countries (Otim et al.).

Ogunseye has enriched this research topic with his study entitled “*Nigerian Results-Based Financing Fellowship: A Strategic Approach for Sustaining Results-Based Financing in Nigeria*.” This particular paper emphasizes the need for its utilization as a strategic approach for the sustenance and expansion of RBF in Nigeria.

The next successful publication was entitled “*Rising Catastrophic Expenditure on Households Due to Tuberculosis: Is India Moving Away From the END-TB Goal?*” Prasad et al. reveal that in India, despite free diagnostic and treatment services offered under the national program, households are exposed to catastrophic financial expenditure due to tuberculosis.

Balkhi et al. have contributed with another surprising paper entitled “*Impact of Healthcare Expenditures on Healthcare Outcomes in the Middle East and North Africa (MENA) Region: A Cross-Country Comparison, 1995–2015*” throughout a complex methodological framework, authors have proven that a large variation was demonstrated between health expenditures per capita and life expectancy in MENA countries, and this variation is growing with time.

Another valuable piece outsourcing from the Sub-Saharan Africa region was entitled “*What Drives Outpatient Care Costs in Kenya? An Analysis With Generalized Estimating Equations*.” It has been discovered that cost of outpatient care changes with age in a sinusoidal manner. Households whose heads reported primary or secondary school education level tended to spend less on outpatient costs than households headed by those who never went to school (Mwenda).

Last but not least, Wu et al. found out in methodologically grounded results that heterogeneous factors influence online timebank nursing for dealing with the increasingly serious population aging problem in China and in Beijing metropolitan area in particular.

This research topic has attempted to partially explain the core challenges of medical care financing and spending primarily across LMICs. Submitted manuscripts have mostly focused on issues relevant to health care economics among the developing world nations. Various health-economic evaluations and health policy analyses have been published outsourcing from academia, industry, and regulatory authorities. How much the editors and authors achieved their goals yet remains to be seen.

## Author Contributions

MJ has prepared the manuscript draft while ZÇ, PF, SM, and MO have revised it for important intellectual content. All authors contributed to the article and approved the submitted version.

## Funding

The authors would like to hereby express gratitude to Grant No. 175014 of the Ministry of Education, Science and Technological Development of the Republic of Serbia, out of which some underlying studies were partially financed. Publication of results was not contingent on Ministry's censorship or approval.

## Conflict of Interest

The authors declare that the research was conducted in the absence of any commercial or financial relationships that could be construed as a potential conflict of interest.

## Publisher's Note

All claims expressed in this article are solely those of the authors and do not necessarily represent those of their affiliated organizations, or those of the publisher, the editors and the reviewers. Any product that may be evaluated in this article, or claim that may be made by its manufacturer, is not guaranteed or endorsed by the publisher.
